# Effectiveness and Safety of Pirfenidone and Nintedanib for Pulmonary Fibrosis in COVID-19-Induced Severe Pneumonia: An Interventional Study

**DOI:** 10.7759/cureus.29435

**Published:** 2022-09-21

**Authors:** Rajkamal Choudhary, Abilesh Kumar, Obaid Ali, Anjum Pervez

**Affiliations:** 1 Department of Medicine, Jawahar Lal Nehru Medical College, Bhagalpur, IND; 2 Dialysis Unit, Jawahar Lal Nehru Medical College, Bhagalpur, IND

**Keywords:** intensive care units, artificial, respiration, pirfenidone, nintedanib, pulmonary fibrosis, respiratory distress syndrome, covid-19

## Abstract

Background

After a diagnosis of two to five years, the survival length for pulmonary fibrosis (PF) is considered to be medium. The primary objective of PF treatment is to stabilize or minimize the pace of progression of the illness. The treatment of PF by nintedanib and pirfenidone was a breakthrough. In a group of coronavirus disease 2019 (COVID-19)-induced PF patients, we examined the efficacy of pirfenidone and nintedanib.

Methodology

From May 2021 to April 2022, 5,000 patients receiving antifibrotic treatment with pirfenidone or nintedanib (mean age of 78.3 ± 23.8) for PF were identified. Their clinical and functional information was retrospectively examined at zero, six, and twelve months of therapy.

Results

The average age of patients receiving nintedanib was greater than the average age of the pirfenidone group (p < 0.0001). Exertional dyspnea and dry cough, with no distinction between the two groups, were the most prevalent symptoms of the illness (p < 0.05). No significant changes between patients on pirfenidone and nintedanib were seen in forced vital capacity, forced expiratory volume in one second, total lung capacity, and diffusing capacity for carbon monoxide at zero or six months (p > 0.05). After one year, lung function measures were similar to the baseline in individuals treated with pirfenidone and nintedanib. This study highlights the appearance of both antifibrotic medicines as promising treatment options for functional stability in COVID-19-induced PF patients.

Conclusions

The patients affected by COVID-19 and undergoing fibrinolytic therapy may be well treated by any of the drugs with a significant improvement.

## Introduction

The coronavirus disease 2019 (COVID-19) pandemic, caused by severe acute respiratory syndrome coronavirus 2 (SARS-CoV-2), has been affecting the entire planet since early 2020 [1]. It has a short (median of three days) [1] period of incubation and is highly transmissible. The disease can present as an asymptomatic infection or as a serious life-threatening infection. Though different systemic characteristics may exist, the common manifestation of pneumonia involves the lower respiratory system which often leads to acute respiratory distress syndrome (ARDS). In certain individuals, multiorgan failure is noted, which may be due to an interaction between cytokine and thrombotic microangiopathy [2].

Early lung illness is pathologically characterized by neutrophil and exudative pulmonary capillaritis with evidence of microthrombosis [2]. Late-stage pneumonia (OP) is developed in the airspaces with an increase in fibroblasts. Clinically, after COVID-19 pneumonia, many patients recover fully. Other people may exhibit signs of recovery from acute sicknesses, such as the resolution of fever and the return of organ function, but they may, nevertheless, continue to display some degree of dyspnea while being chronically infiltrated with radiologic trials and/or hypoxemia. These patients often have patchy, multifocal consolidation and floor-ground opacities, which might suggest lung-related surgery on computed tomography (CT). Coarse cross-linking and parenchymal bands can also be noted. Glucocorticoids are frequently used for the treatment of patients with diffuse pulmonary diseases post-COVID-19. Even though the majority of patients who had a primary OP damage pattern either resolved their lung parenchymal abnormalities on their own or with the help of glucocorticoids, there is still a possibility that some of these patients could develop signs of pulmonary fibrosis (PF) in the form of traction and/or honey combining. Some individuals have continuing respiratory problems after steroid therapy and can be shown to have persisting reticulation or a non-resolution accumulation of early fibrosis chest imaging.

Pirfenidone and nintedanib have proven to be efficient in the treatment of PF. Moreover, nintedanib has been shown to be beneficial in treating systemic interstitial lung disease (ILD) and progressive fibrous ILDs associated with non-interstitial pulmonary fibrosis (IPF) etiology. It is also uncertain if these medicines would treat post-COVID-19 lung fibrosis effectively. Because the ultimate routes of lung fibrosis among many diffuse parenchymal lung disorders (DPLDs) are similar, these antifibrotic medicines are perhaps useful for post-COVID-19 fibrosis. Regarding the role of pirfenidone or nintedanib in COVID-19, there are no randomized controlled trials in the literature. In this study, we sought to examine the effectiveness and safety of pirfenidone in the treatment of post-COVID-19 lung fibrosis and compared it to nintedanib.

## Materials and methods

From May 2021 to April 2022, antifibrotic treatment with pirfenidone or nintedanib was started in 5,000 COVID-19-induced PF patients. Ethical approval was obtained by Jawaharlal Nehru Medical College & Hospital Ethical Committee (ref/2020/1695). Demographic data, familial histories of lung fibrosis, smoking, occupational/environmental exposure, and comorbidities were used to assess clinical and functional features. Age, sex, bronchoalveolar lavage (BAL) differential cell count, BAL lymphocyte phenotype, lung function tests, oxygen desaturation during a six-minute walking test, and partial pressure of oxygen (PaO_2_) values measuring blood gas analysis were recorded. These parameters were reported. In addition, functional, radiological, histological, and immunological data were recorded. Pulmonary function parameter testing was done in accordance with the anticipated percent forced expiratory volume in one second (FEV1), forced vital capacity (FVC), total lung capacity (TLC), and diffusing capacity for carbon monoxide (DLCO) recommendations of the American Thoracic Society and European Respiratory Society (ATS/ERS) guidelines. In 70/82 IPF patients, IPF was diagnosed based on clinical radio-specific criteria. Chest X-rays were obtained for all patients in posterior and anterior and lateral projections, along with a high-resolution computerized tomography (CT) of the chest. In the framework of an interdisciplinary conference, a diagnosis was formulated. Inclusion criteria include age above 18 years. COVID-19 was diagnosed based on a test conducted on a respiratory sample (upper or lower respiratory) or immunoglobulin M (IgM) antibody positive or a quick antigen test with consistent clinicoradiologic results in the past four months using a real-time transcription chain polymerase reaction test. In addition, we included patients with persistent respiratory symptoms, post-COVID-19 parenchymal involvement >10% of the lung parenchyma during visual inspection of the scans with ray signs of fibrosis, or persistent reticulation or consolidation despite a test of the use of glucocorticoid (minimum 10 mg/day prednisolone or equivalent). Traction bronchiectasis or reduced volume of the lung. While exclusion criteria included pregnant or lactating women. Patients with known pirfenidone or nintedanib contraindications (advanced liver cirrhosis, chronic hepatic transaminase elevation, proven pirfenidone or nintedanib hypersensitivity, anticoagulant or aspirin in high dose, or stent in situ). Patients with diffuse lung disease prior to the diagnosis of COVID-19. The outcome measures included changes in the FVC, FEV1, TLC, and DLCO.

For pirfenidone, a dosage of 600 mg/day was initiated. The dose was increased to a target level of 2,400 mg/day by 600 mg/day each three to seven days. The highest dose tolerated for a total duration of 24 weeks was delivered to patients after randomization.

For nintedanib, a dosage of 150 mg twice daily was provided to subjects in this group. The tests for liver function were monitored as above. If intolerance was achieved to the dosage of 300 mg/day, the dose was decreased to 100 mg twice a day.

All data were reported as mean and standard deviation (SD). In both treatment groups, the Wilcoxon test was employed to describe the trend. Variance analysis for comparing groups was conducted. Statistical significance was defined as p-values of <0.05. Data analysis was done using SPSS version 21 (IBM Corp., Armonk, NY, USA).

## Results

In this study, 5,000 COVID-19-induced PF patients were included; 2500 were treated with pirfenidone, and 2,500 were treated with nintedanib.

The average age of people who were administered pirfenidone was 71.04 ± 6.1 and of those treated with nintedanib was 78,33 ± 5.5 years. Due to the criteria for inclusion in the pirfenidone group, the median age of the patients treated for nintedanib was considerably greater than that in the other group (p < 0.0001). In our PF population, there was an obvious prevalence (80%) of men without any variation across groups. Overall, 72% of the patients in the pirfenidone group were recognized as being smokers, while only 50% of smokers in the nintedanib group were identified. The FVC, FEV1, DLCO, and FLC for lung function in patients treated with pirfenidone in the IPF group at zero and six and 12 months were 78.7 ± 16.9%, 83.3 ± 16.5%, 51.2 ± 15.3%, and 68.7 ± 14.7%, respectively. The parameters for the lung function test in the population of nintedanib-treated patients at zero and six and 12 months were 80.9% ± 19.6% for the FVC, and the FEV1, the DLCO, and TLC were 87 ± 19%, 47 ± 15%, and 66.3 ± 13%, respectively. No substantial changes were detected in the percentages of FVC, FEV1, TLC, and DLCO between two medications (p = 0.59, p = 0.37, p = 0.21, p = 0.48, respectively).

**Figure 1:**
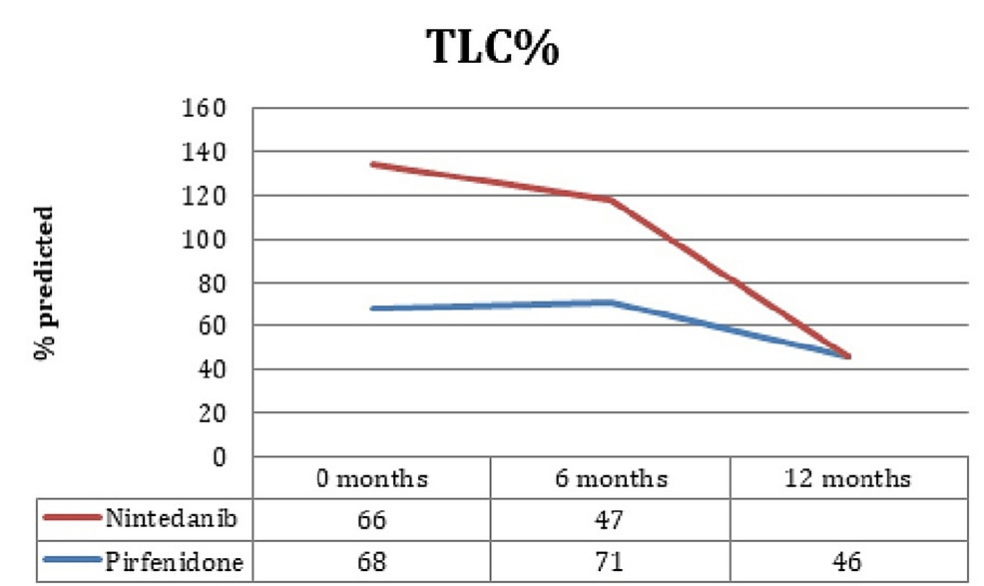
TLC values for the groups. TLC: total lung capacity

**Figure 2:**
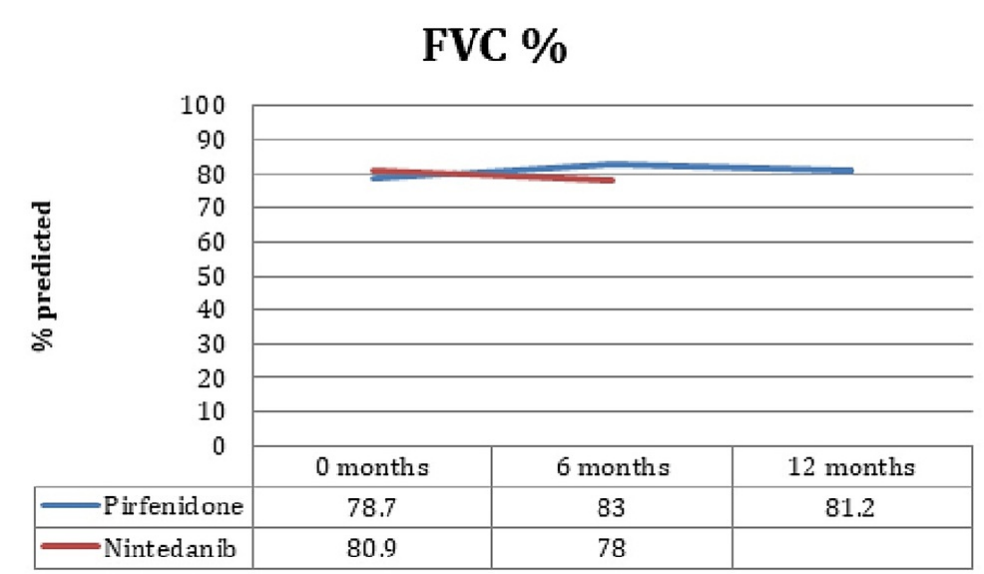
FVC values in the groups. FVC: forced vital capacity

**Figure 3:**
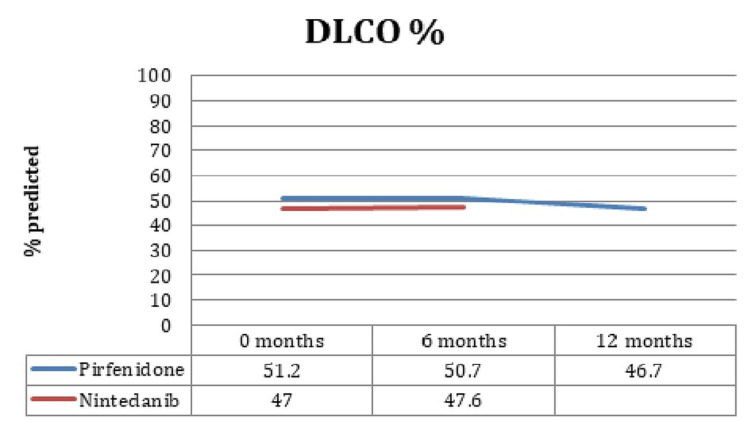
DLCO values in the groups. DLCO: diffusing capacity for carbon monoxide

FVC, FEV1, DLCO, and TLC prediction for the following six months was 83 ± 17.3%, 88.6 ± 18.3%, 50.7 ± 13.3%, and 71.3 ± 15%, respectively, in pirfenidone patients, while in the six-month follow-up, the prediction was 84 ± 13.8%, 67.15 ± 16%, 78.3 ± 13.3%, and 50.3 ± 15% in patients treated with pirfenidone. It is interesting that no significant changes in FVC, FEV1, DLCO, or TLC were identified during the six-month follow-up (p = 0.54, p = 0.38, p = 0.76, p = 0.31, respectively) between the patients treated with pirfenidone and nintedanib. Figure 1-3 elaborates the values in chart form.

## Discussion

In the majority of PF patients, pirfenidone exhibited a satisfactory tolerability profile. Gastrointestinal problems and skin responses were treatable. In our population, the adverse event that occurred most frequently was nausea and dyspepsia, accounting for 35% of cases, followed by diarrhea (28% of cases), and skin rash (19% of cases). The development of prokinetic drugs and proton-pump inhibitors has made it possible to utilize broad-spectrum sunscreeincreasedncrease in the number of people who experience unfavorable gastrointestinal effects. Three individuals had a serious rash and photosensitivity resulting from continuous exposure to the sun for occupational reasons.

Therapy with nintedanib was well tolerated. Diarrhea was the most prevalent adverse effect in 40% of patients and premature cessation was required in 18% of cases. Only 5% of patients had to be interrupted, and the remaining subjects improved from 150 to 100 mg twice a day following the dosage decrease. Altered liver enzymes were reversible following a dosage decrease in 8% of individuals treated with this medicine. In four individuals, the rise in aminotransferase levels was not reversible with changes and cessation after six months of treatment and liver modification. Both were secondary to lithiasis and persistently mild liver impairment. Several worldwide clinical trials have reported the safety and effectiveness of pirfenidone and nintedanib treatment in PF patients [3-6]. There is currently very little available dahe actual experience of antifibrotic medicines in patients with PF [7-9]. This research examined the efficacy of a COVID-19-diagnosed PF population with pirfenidone and nintedanib treatment. This research provides information on medication efficacy in the real clinic of a university hospital but can be only compared in part with international clinical trials, with a 12-month follow-up limit for patients treated with nintedanib. There has been a predicted high incidence among males compared to females, with PF characteristics more common in males aged more than 65 years. However, ILD linked with connective tissue disease is more frequent in women [10]. Patients in the nintedanib subgroup were older than in the pirfenidone group, given that only nintedanib and not pirfenidone can be administered to PF patients above the age of 80. Bi-basal crackling and a restricted functioning deficit were seen in all patients (100%), with a significant drop in DLCO at the beginning of the illness [11,12]. With six months of follow-up, the two medicines seemed to stabilize their spirometric profile, generally associated with lung function decline, over the duration of a couple of months. Stabilizations in the pirfenidone group were also confirmed in line with literature findings at 12 months. Regardless of their fundamental functional status, our real-life research coupled the two medications with functional improvements comparable to those documented in clinical trials and with lowered FVC rates in a similar manner. In the older group of patients treated with nintedanib, stabilization of pulmonary indices has also been reported [13-15].

In this pilot investigation to evaluate the benefits of pirfenidone and nintedanib treatment in a PF cohort of Indians, the stability of the pulmonary function testing parameters was demonstrated. Antifibrotic medications were also shown to functionally stabilize elderly individuals with PF.

## Conclusions

A unique therapeutic strategy for treating COVID-19-induced lung fibrosis may be offered by nintedanib and pirfenidone. Post-COVID-19 PF is evolving into a deadly threat to global health given the scope of the COVID-19 pandemic and the number of people requiring invasive ventilation globally. With the use of nintedanib and pirfenidone, our patients saw a considerable improvement in clinical indicators.

These medications may be able to treat severe COVID-19 and reduce the risk of lung fibrosis. Trials examining the effectiveness and safety of nintedanib and pirfenidone against COVID-19-related fibrotic lung consequences are required to address this new problem. Patients affected by COVID-19 and undergoing fibrinolytic therapy can be well treated by any of the drugs with a significant improvement.
